# Supplementary material for: A Global Screen for Assembly State Changes of the Mitotic Proteome by SEC-SWATH-MS
Source: Cell Syst. 2020 Feb 26;10(2):133–155.e6. doi: 10.1016/j.cels.2020.01.001 (PMC7042714; doi:10.1016/j.cels.2020.01.001)

Q6UX71 | PXDC2_HUMAN | PLXDC2 TEM7R UNQ2514/PRO6003
Monomer MW [kDa]: 59.583 Monomer expected elution fraction: 45

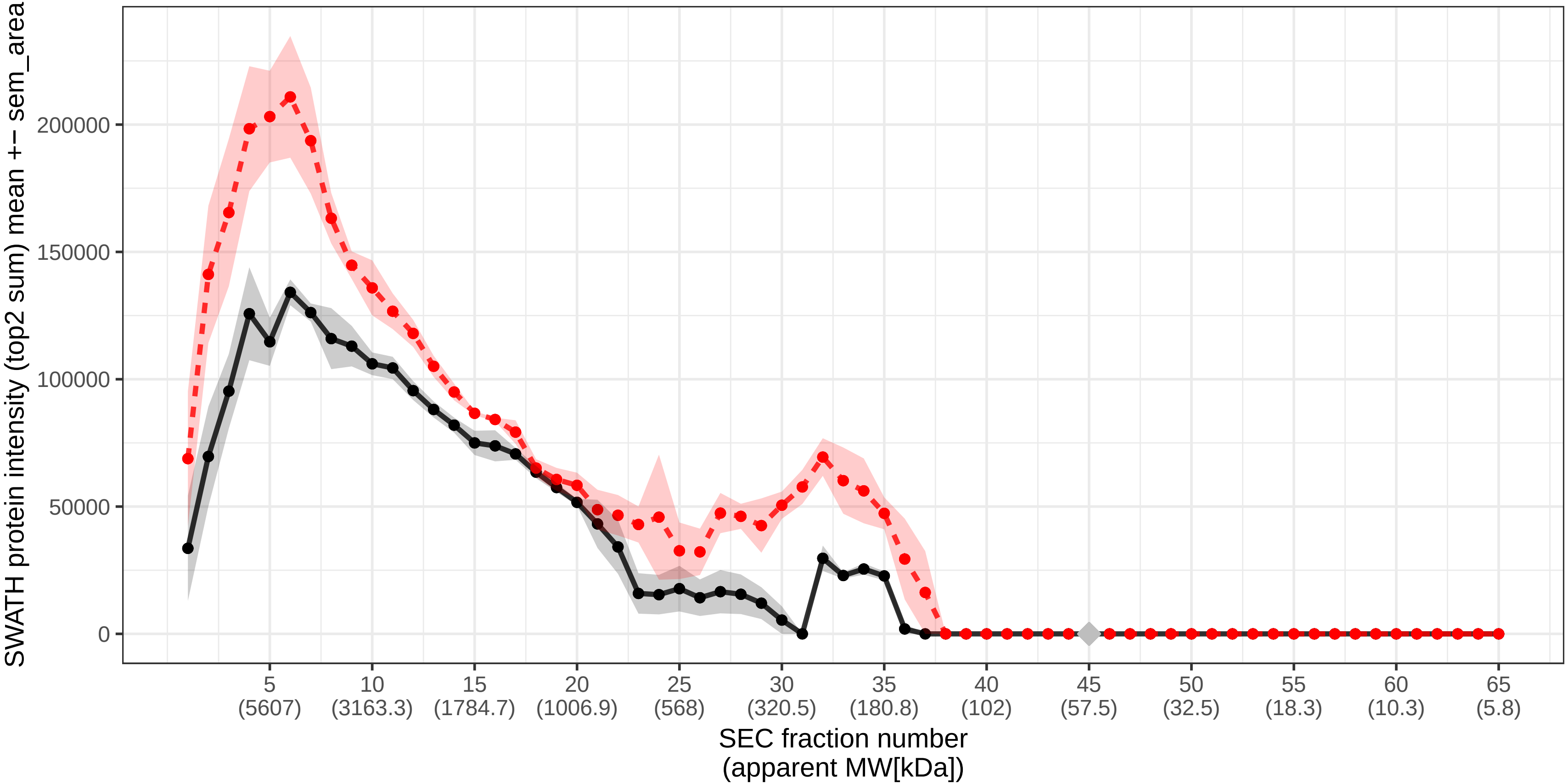

Supplement: Data S1. SEC-SWATH-MS Protein Chromatograms, Related to Figure 1 [file mmc6.zip › SECchrom_Q6UX71_PXDC2_HUMAN_PLXDC2_TEM7R_UNQ2514_PRO6003.pdf]
